# Survival and prognostic analysis of preoperative inflammatory markers in patients undergoing surgical resection for laryngeal squamous cell carcinoma

**DOI:** 10.1186/s12885-018-4730-x

**Published:** 2018-08-13

**Authors:** Linyan Chen, Hao Zeng, Jiapeng Yang, Yuqing Lu, Dan Zhang, Jinggan Wang, Chienyun Kuang, Sha Zhu, Manni Wang, Xuelei Ma

**Affiliations:** 1 Department of Biotherapy, Cancer Center, State Key Laboratory of Biotherapy, West China Hospital, Sichuan University and Collaborative Innovation Center, No.37, Guoxue Alley, Chengdu, 610041 People’s Republic of China; 2 The People’s Hospital of Hechi, Hechi, Guangxi People’s Republic of China; 3 West China School of Stomatology, Sichuan University, Chengdu, People’s Republic of China

**Keywords:** Laryngeal cancer, Neutrophil, Lymphocyte, Platelet, Monocyte, Prognosis

## Abstract

**Background:**

To estimate the prognostic value of inflammatory markers in patients with laryngeal squamous cell carcinoma (LSCC).

**Methods:**

A total of 361 resected LSCC patients were included. The preoperative and postoperative neutrophil-to-lymphocyte ratio (NLR), platelet-to-lymphocyte ratio (PLR), monocyte-to-lymphocyte ratio (MLR), alkaline phosphatase (ALP) and l actate dehydrogenase (LDH) were assessed. The Kaplan-Meier survival analysis and Cox regression analysis were conducted on overall survival (OS) and progression-free survival (PFS).

**Results:**

Both Kaplan-Meier analysis and univariate analysis demonstrated significant prognostic value of preoperative and postoperative NLR, PLR and MLR. However, only preoperative ALP was predictive of OS and PFS, and LDH failed to be predictor of OS and PFS. The multivariate analysis showed that preoperative NLR (OS: HR = 1.64, 95%CI: 1.06–2.54, *p* = 0.026; PFS: HR = 1.52, 95%CI: 1.04–2.23, *p* = 0.029) and postoperative MLR (OS: HR = 2.02, 95%CI: 1.29–3.14, *p* = 0.002; PFS: HR = 1.57, 95%CI: 1.05–2.34, *p* = 0.026) were independently related with survival.

**Conclusions:**

The elevated preoperative NLR, PLR, MLR and ALP were significantly associated with worse survival and cancer progression. The preoperative NLR and postoperative MLR might be independent prognostic markers of OS and PFS in LSCC patients undergoing surgical resection.

**Electronic supplementary material:**

The online version of this article (10.1186/s12885-018-4730-x) contains supplementary material, which is available to authorized users.

## Background

Laryngeal cancer is one of the most common cancers in head and neck region with 26,300 new cases and 14,500 deaths in China in 2015 [1]. The 5-year overall survival rate was approximately 60% in the United States based on SEER Cancer Statistics Review. Laryngeal squamous cell carcinoma (LSCC) accounts for 85–95% of laryngeal cancer [2]. Various treatments are used to cure laryngeal cancer patients, including surgery, radiotherapy and chemotherapy [3]. However, the review of American Cancer Society suggested that 5-year survival rate was on the decline despite the improvement of therapy [4, 5]. Therefore, it is of great importance to estimate the effectiveness of novel prognostic markers for predicting survival and optimizing therapeutic strategies in LSCC patients.

Pathological TNM stage was considered as the best predictor of long-term survival [6], but it is not available before surgery. Recent evidences have proved that tumor-related immune responses are significantly related with tumor progression [7]. Cytokines produced by tumor cells or tumor microenvironment can stimulate the host inflammation, which led to a rise of circulating peripheral blood cells, including neutrophil, lymphocyte, platelet and monocyte [8]. The neutrophil-to-lymphocyte ratio (NLR) was related with tumorigenesis, aggressiveness and poor prognosis [9]. Meanwhile, the platelets can promote the angiogenesis, microvascular permeability and extravasation of tumor cells [10]. Accordingly, the NLR and platelet-to-lymphocyte ratio (PLR) have reflected significant prognostic role in different cancers, including prostate cancer and lung cancer [11, 12]. And NLR was regarded as a better predictor than PLR in several studies [13]. The monocyte-to-lymphocyte ratio (MLR), alkaline phosphatase (ALP) and lactate dehydrogenase (LDH) were also reported as prognostic factors [14]. In terms of LSCC, high preoperative NLR and PLR were significantly relevant with worse OS and PFS in LSCC patients [10, 15, 16]. However, the independent prognostic value of NLR and PLR based on multivariate analysis still remains controversial. The MLR, LDH and ALP have not been systematically estimated, which need further analysis and discussion.

The aim of this study was to estimate and compare the prognostic value of inflammatory markers (NLR, PLR, MLR, ALP and LDH) on overall survival (OS) and progression-free survival (PFS) in LSCC patients treated with surgical resection. Therefore, we performed a retrospective and single-center study with a large sample of 361 eligible patients.

## Methods

### Patients

A total of 361 patients diagnosed with LSCC in the West China Hospital (Sichuan, China) from January 2010 to December 2014 were included in the present study. Patients were included refers to the following criteria: (1) LSCC was confirmed by pathological examination; (2) patients were older than 18 years old; (3) patients underwent laryngectomy without neoadjuvant chemotherapy or radiotherapy; (4) clinical information such as laboratory test and radiologic examination was complete; (5) the minimum follow-up period was 3 years; and (6) patients had no concurrent acute inflammatory diseases or haematological disorders that might affect the level of inflammatory markers.

### Data extraction and follow-up

We extracted the data of patient demographics, laboratory tests, imaging reports and pathologic characteristics from electronic medical records. The TNM stage of laryngeal cancer was confirmed by pathological findings primarily and imaging reports secondly according to AJCC- TNM stage seventh edition [17]. The NLR, PLR, MLR, ALP and LDH were obtained from blood tests within 1 week before surgery (preoperative), and 2 week to 1 month after surgery but before adjuvant therapy (postoperative). Patients were evaluated through routine radiologic and laboratory reexamination and telephone follow-up. The median period of follow-up was 47 months (range: 4–98). The endpoints were OS and PFS. The OS was measured from the date of pathologic diagnosis to death, and the PFS referred to the period from pathologic diagnosis to the date of recurrence, metastasis or death. In addition, the living patients without cancer progression were censored on the last follow-up. The patients were followed up until December 2017 or their death.

### Statistical analysis

The differences between patients grouped by high and low level of preoperative NLR PLR and MLR were evaluated by Chi squared test. The t-test and 1-way analysis of variance (ANOVA) were used to compare the mean value of preoperative inflammatory markers. After classifying the patients with cancer progression, we calculated the optimal cutoff values of NLR, PLR, MLR, ALP and LDH based on maximum Youden index (sensitivity+specificity− 1) through receiver operating curve (ROC) analysis. Survival curves were obtained by Kaplan-Meier survival analysis and compared by log-rank test. Both univariate and multivariate Cox proportional hazard regression models were used to estimate the association between inflammatory markers and survival outcome. The significant variables in univariate analysis or Chi squared test were included in multivariate analysis. The multivariable analysis was conducted through forward stepwise (Likelihood Ratio) selection, thus only the significant variables were present with hazard ratios (HRs) and 95% confidence intervals (CIs). We performed all the statistical analyses on SPSS version 21.0 and *p* <  0.05 was defined as statistically significant.

## Results

### Cutoff values of inflammatory markers

According to the ROC curves, the areas under the curve (AUCs) and 95%CIs of preoperative NLR, PLR, MLR, ALP and LDH were 0.631 (0.569–0.692, *P* <  0.001), 0.612 (0.543–0.680, *P* = 0.002), 0.573 (0.514–0.632, *P* = 0.017), 0.563 (0.495–0.631, *P* = 0.074) and 0.493 (0.429–0.558, *P* = 0.841), respectively. The optimal cutoff value was 2.45 for NLR, 114 for PLR, 0.21 for MLR, 78 (U/L) for ALP and 185 (U/L) for LDH. In the same way, the AUCs and 95%CI of postoperative NLR, PLR, MLR, ALP and LDH were 0.568 (0.509–0.627, *P* = 0.025), 0.605 (0.542–0.688, *P* = 0.001), 0.614 (0.550–0.678, *P* <  0.001), 0.495 (0.423–0.568, *P* = 0.895) and 0.524 (0.460–0.587, *P* = 0.466), respectively. The optimal cutoff value was 2.85 for NLR, 111 for PLR, 0.36 for MLR, 84 (U/L) for ALP and 158 (U/L) for LDH.

### Baseline characteristics of patients

The entire cohort (*N* = 361) consisted of 353 male and 8 female with median age at diagnosis of 60 years old (range: 35–87). The detailed process of patient selection was presented in Additional file 1: Figure S1. All patients were diagnosed with laryngeal squamous cell carcinoma without distant metastases. The postoperative therapies included intensity-modulated radiation therapy and cisplatin based chemotherapy. There were 62 and 6 patients received radiotherapy or chemotherapy alone, and 20 patients underwent both chemotherapy and radiotherapy. Forty-seven patients had medications that might affect inflammatory markers within 1 week before blood test. A total of 87 patients had comorbidities, 51, 20 and 6 patients had hypertension, diabetes and hyperlipemia. In addition, 14, 5 and 3 patients had chronic obstructive pulmonary disease, coronary heart disease and gastritis.

The grouped clinicopathological parameters according to NLR, PLR and MLR were showed in Table 1. Significant differences were found in the distribution of tumor location and histology between high and low PLR groups. Compared with low NLR/PLR group, there were more patients with worse T classification and TNM stage in the high NLR/PLR group. The NLR, PLR and MLR were all significantly relevant with selection of surgical removal. We also compared the mean value of preoperative inflammatory markers by patient’s clinicopathological characteristics (Additional file 2: Table S1). The adverse tumor features including worse T classification, advanced TNM stage and total laryngectomy were associated with higher level of inflammatory markers.

**Table 1:** Correlation between preoperative NLR, PLR, MLR and baseline characteristics of patients

Variables	Parameters	*n*	NLR		*p*	PLR		*p*	MLR		*p*
			< 2.45	≥ 2.45		< 114	≥ 114		< 0.21	≥ 0.21	
Age (year)	< 60	167	111	56	0.543	115	52	0.638	93	74	0.326
	≥ 60	194	123	71		138	56		98	96	
Gender	Male	353	229	124	0.814	248	105	0.934	185	168	0.364
	Female	8	5	3		5	3		6	2	
Tumor location	Supraglottic	280	190	90	0.080	204	76	0.025*	149	131	0.959
	Glottic	70	38	32		40	30		36	34	
	Subglottic	11	6	5		9	2		6	5	
T classification	T1	115	85	30	0.001*	87	28	0.006*	68	47	0.148
	T2	126	88	38		93	33		68	58	
	T3	68	38	30		47	21		34	34	
	T4	52	23	29		26	26		21	31	
N classification	N0	320	210	110	0.416	229	91	0.221	174	146	0.081
	N1	28	15	13		16	12		14	14	
	N2	13	9	4		8	5		3	10	
TNM stage	I	114	85	29	0.002*	87	27	0.002*	68	46	0.094
	II	118	81	37		88	30		64	54	
	III	71	40	31		49	22		36	35	
	IV	58	28	30		29	29		23	35	
Histology	Well	135	95	40	0.021	101	34	0.049*	73	62	0.205
	Moderate	159	105	54		113	46		89	70	
	Poor	67	34	33		39	28		29	38	
Laryngectomy	Partial	287	202	85	< 0.001*	216	71	< 0.001*	161	126	0.017*
	Total	74	32	42		37	37		30	44	
Radiotherapy	No	279	180	99	0.824	198	81	0.498	143	136	0.245
	Yes	82	54	28		55	27		48	34	
Chemotherapy	No	335	222	113	0.039*	237	98	0.323	177	158	0.921
	Yes	26	12	14		16	10		14	12	
Medication	No	314	205	99	0.016*	221	93	0.748	166	148	0.967
	Yes	47	29	28		32	15		25	22	
Comorbidities	No	274	175	109	0.014*	190	84	0.586	145	129	0.994
	Yes	87	59	18		63	24		46	41	

*Abbreviations*: *NLR* neutrophil-lymphocyte ratio, *PLR* platelet-lymphocyte ratio, *MLR* monocyte-lymphocyte ratio

*Statistically significant *p* < 0.05

### Overall survival according to preoperative inflammatory markers

**Fig. 1:**
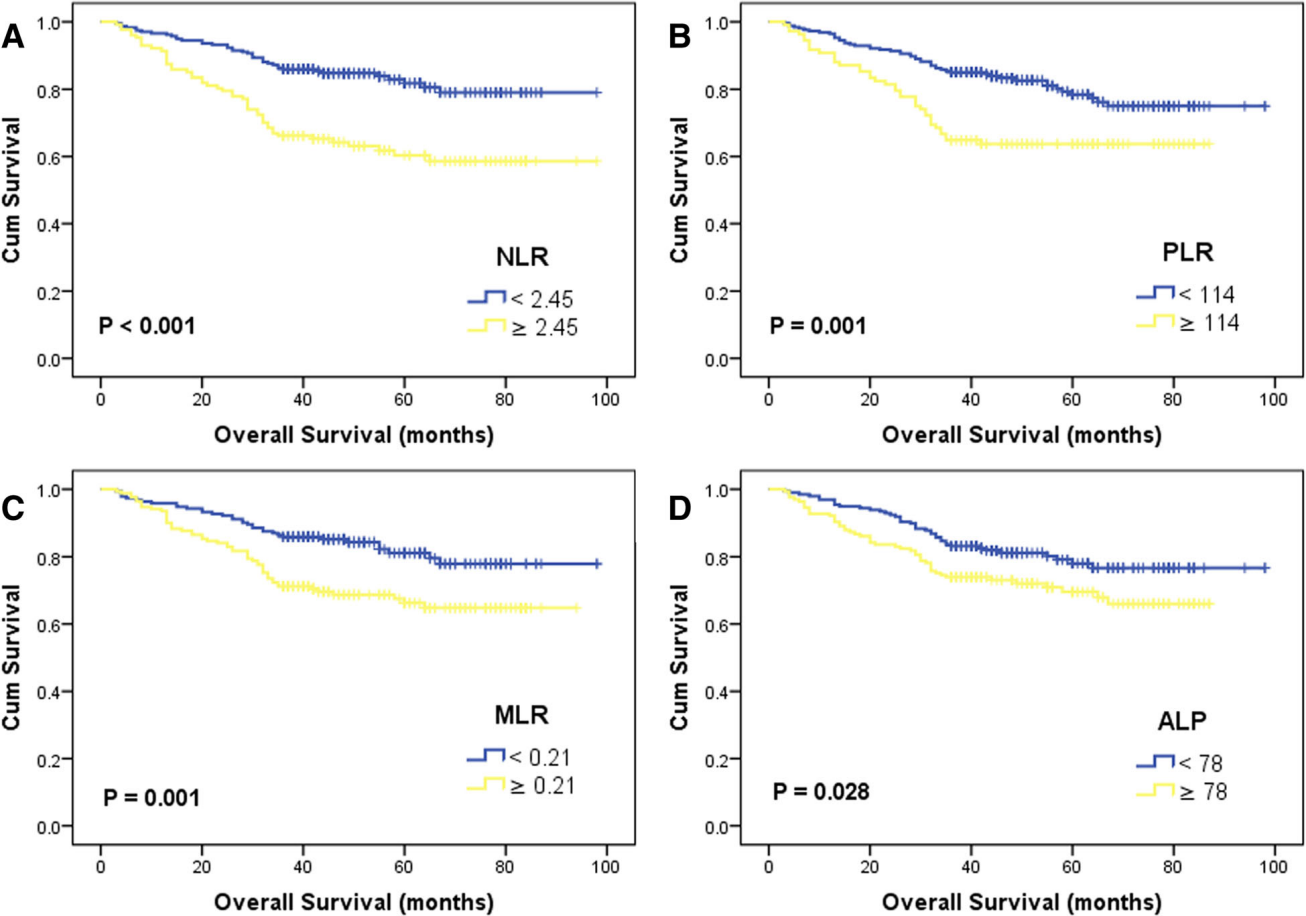
Kaplan-Meier survival curves for preoperative NLR **a** PLR **b** MLR **c** ALP **d** on overall survival

On the last follow-up, 40 (17.1%) and 49 (42.5%) patients were died in the NLR <  2.45 and NLR ≥ 2.45 groups, the 5-year OS rates were 81.8% and 60.3% (*p* <  0.001). The univariate HR and 95% CI of NLR was 2.53 (1.66–3.84, *p* <  0.001). The survival results were also significantly different between the PLR (HR = 2.05, 95% CI: 1.35–3.12, *p* = 0.001), MLR (HR = 1.99, 95% CI: 1.30–3.06, *p* = 0.002) and ALP (HR = 1.59, 95% CI: 1.05–2.41, *p* = 0.030) groups (Fig. 1, Table 2). However, the LDH failed to show significant prognostic role.

**Table 2:** Univariate analysis of inflammatory markers associated with survival

Variables	Parameters	*n*	Overall survival (%)					Progression-free survival (%)				
			3-year	5-year	*p* ^a^	HR (95% CI)	*p* ^b^	3-year	5-year	*p* ^a^	HR (95% CI)	*p* ^b^
Overall	–	361	78.9	74.1	–			69.1	65.5	–		
Preoperative												
NLR	< 2.45	234	85.9	81.8		1		76.4	73.8		1	
	≥ 2.45	127	66.1	60.3	< 0.001*	2.53 (1.66–3.84)	< 0.001*	55.6	50.9	< 0.001*	2.13 (1.49–3.07)	< 0.001*
PLR	< 114	253	85.0	78.4		1		73.8	69.3		1	
	≥ 114	108	64.8	63.7	0.001*	2.05 (1.35–3.12)	0.001*	58.3	57.0	0.015*	1.58 (1.09–2.29)	0.016*
MLR	< 0.21	191	85.9	81.1		1		75.3	71.3		1	
	≥ 0.21	170	71.2	66.2	0.001*	1.99 (1.30–3.06)	0.002*	61.8	58.8	0.010*	1.61 (1.12–2.32)	0.011*
ALP	< 78	196	83.2	78.0		1		74.0	69.6		1	
	≥ 78	165	73.9	69.6	0.028*	1.59 (1.05–2.41)	0.030*	63.2	60.8	0.032*	1.48 (1.03–2.13)	0.034*
LDH	< 185	258	79.8	75.6		1		70.2	66.7		1	
	≥ 185	103	76.7	70.6	0.275	1.28 (0.82–1.98)	0.278	66.1	64.5	0.451	1.16 (0.79–1.71)	0.453
Postoperative												
NLR	< 2.85	231	88.7	82.8		1		77.7	72.9		1	
	≥ 2.85	130	61.5	58.6	< 0.001*	3.18 (2.08–4.86)	< 0.001*	53.7	52.5	< 0.001*	2.18 (1.52–3.14)	< 0.001*
PLR	< 111	191	89.5	85.9		1		78.3	72.3		1	
	≥ 111	170	67.1	61.0	< 0.001*	3.13 (1.98–4.94)	< 0.001*	58.9	58.0	0.001*	1.85 (1.28–2.68)	0.001*
MLR	< 0.36	280	86.4	80.8		1		75.8	71.8		1	
	≥ 0.36	81	53.1	51.3	< 0.001*	3.65 (2.40–5.56)	< 0.001*	45.0	43.2	< 0.001*	2.60 (1.79–3.79)	< 0.001*
ALP	< 84	237	79.7	74.1		1		70.2	65.8		1	
	≥ 84	124	77.4	74.7	0.694	1.09 (0.71–1.69)	0.695	67.0	65.5	0.509	1.14 (0.78–1.66)	0.512
LDH	< 158	135	82.2	79.7		1		73.9	69.8		1	
	≥ 158	226	77.0	70.8	0.073	1.51 (0.96–2.39)	0.076	66.1	63.0	0.097	1.39 (0.94–2.05)	0.100

*Abbreviations*: *HR* hazard ratio, *CI* confidence interval, *NLR* neutrophil-lymphocyte ratio, *PLR* platelet-lymphocyte ratio, *MLR* monocyte-lymphocyte ratio, *ALP* alkaline phosphatase, *LDH* lactate dehydrogenase

^*^Statistically significant *p* < 0.05

^a^the *p* value for log-rank test ^b^ the *p* value for HR in Cox analysis

In terms of clinicopathological parameters, the patient’s age (*p* = 0.009), tumor location (all *p* <  0.001), T classification (*p* < 0.001), N classification (*p* < 0.001), TNM stage (*p* < 0.001), histology (*p* < 0.001) and laryngectomy methods (*p* < 0.001) were found statistically significant in univariate analysis (Table 3). However, the multivariate analysis only supported that NLR (HR = 1.64, 95%CI: 1.06–2.54, *p* = 0.026), tumor location (glottic vs. supraglottic, HR = 2.71, 95%CI: 1.26–5.80, *P* = 0.011) and TNM stage (HR = 4.24, 95%CI: 2.13–7.79, *p* < 0.001) were independent prognostic factors of OS in LSCC patients (Table 4).

**Table 3:** Univariate analysis of clinicopathological characteristics associated with survival

Variables	Parameters	*n*	Overall survival (%)					Progression-free survival (%)				
			3-year	5-year	*p* ^a^	HR (95% CI)	*p* ^b^	3-year	5-year	*p* ^a^	HR (95% CI)	*p* ^b^
Age (year)	< 60	167	83.8	81.1		1		75.4	72.6		1	
	≥ 60	194	74.7	68.2	0.008*	1.80 (1.16–2.79)	0.009*	63.6	59.5	0.019*	1.56 (1.07–2.28)	0.020*
Gender	Female	8	75.0	35.7		1		62.5	–		1	
	Male	353	79.0	74.1	0.372	0.60 (0.19–1.89)	0.379	69.2	65.7	0.576	0.72 (0.23–2.28)	0.580
Tumor location	Glottic	280	83.9	79.5		1		74.1	70.2		1	
	Supraglottic	70	65.7	60.5		2.33 (1.46–3.70)	< 0.001*	57.3	54.9		1.77 (1.16–2.70)	0.008*
	Subglottic	11	36.4	24.2	< 0.001*	5.53 (2.62–11.69)	< 0.001*	12.1	–	< 0.001*	4.50 (2.24–9.03)	< 0.001*
T classification	T1-T2	241	92.1	87.4		1		83.0	79.4		1	
	T3-T4	120	52.5	47.7	< 0.001*	5.15 (2.89–8.72)	< 0.001*	43.2	39.8	< 0.001*	4.09 (2.81–5.95)	< 0.001*
N classification	N0	320	84.1	79.1		1		74.1	70.1		1	
	N1-N2	41	39.0	35.8	< 0.001*	4.32 (2.73–6.84)	< 0.001*	31.7	31.7	< 0.001*	3.13 (2.05–4.79)	< 0.001*
TNM stage	I-II	232	94.0	89.0		1		84.6	80.9		1	
	III-IV	129	51.9	47.5	< 0.001*	6.56 (3.63–9.35)	< 0.001*	43.3	40.1	< 0.001*	4.46 (3.04–6.56)	< 0.001*
Histology	Well-moderate	294	85.4	80.5		1		75.23	71.5		1	
	Poor	67	50.7	46.4	< 0.001*	3.49 (2.28–5.34)	< 0.001*	43.1	40.9	< 0.001*	2.69 (1.83–3.95)	< 0.001*
Laryngectomy	Partial	287	87.8	82.2		1		77.6	72.7		1	
	Total	74	44.6	39.6	< 0.001*	5.06 (3.33–7.68)	< 0.001*	38.1	34.3	< 0.001*	3.22 (2.22–4.67)	< 0.001*
Adjuvant therapy	No	273	79.9	75.0		1		70.2	66.5		1	
	Yes	88	76.1	71.4	0.539	1.16 (0.72–1.87)	0.541	65.5	63.0	0.283	1.25 (0.83–1.89)	0.286
Medication	No	314	79.0	73.7		1		68.1	65.0		1	
	Yes	47	78.7	76.1	0.863	0.95 (0.50–1.78)	0.864	74.4	68.7	0.590	0.86 (0.49–1.50)	0.592
Comorbidities	No	274	78.1	72.8		1		68.0	65.6		1	
	Yes	87	81.6	78.5	0.340	0.77 (0.47–1.31)	0.342	72.6	63.7	0.764	0.94 (0.61–1.45)	0.765

^*^Statistically significant *p* < 0.05

^a^the *p* value for log-rank test ^b^ the *p* value for HR in Cox analysis

**Table 4:** Multivariate analysis of factors associated with survival

Variables	Parameters	Overall survival		Progression-free survival	
		HR (95% CI)	*p*	HR (95% CI)	*p*
Age (year)	< 60 vs. ≥ 60	–	0.068	–	0.107
Tumor location	Glottic vs. Supraglottic	2.71 (1.26–5.80)	0.011*	–	0.445
	Glottic vs. Subglottic	–	0.110	–	0.440
T classification	T1-T2 vs. T3-T4	–	0.135	–	0.593
N classification	N0 vs. N1-N2	–	0.116	–	0.240
TNM stage	I-II vs. III-IV	4.24 (2.13–7.79)	< 0.001*	3.41 (2.25–5.16)	< 0.001*
Histology	Well-moderate vs. Poor	–	0.100	–	0.181
Laryngectomy	Partial vs. Total	–	0.229	–	0.752
Medication	No vs. Yes	–	0.361	–	0.850
Comorbidities	No vs. Yes	–	0.552	–	0.678
Pre NLR	< 2.45 vs. ≥ 2.45	1.64 (1.06–2.54)	0.026*	1.52 (1.04–2.23)	0.029*
Pre PLR	< 114 vs. ≥ 114	–	0.765	–	0.986
Pre MLR	< 0.21 vs. ≥ 0.21	–	0.317	–	0.567
Pre ALP	< 78 vs. ≥ 78	–	0.340	–	0.271
Post NLR	< 2.85 vs. ≥ 2.85	–	0.245	–	0.701
Post PLR	< 111 vs. ≥ 111	–	0.584	–	0.571
Post MLR	< 0.36 vs. ≥ 0.36	2.02 (1.29–3.14)	0.002*	1.57 (1.05–2.34)	0.026*

*Abbreviations*: *HR* hazard ratio, *CI* confidence interval, Pre preoperative, Post postoperative, *NLR* neutrophil-lymphocyte ratio, *PLR* platelet-lymphocyte ratio, *MLR* monocyte-lymphocyte ratio, *ALP* alkaline phosphatase

^*^Statistically significant *p* < 0.05

### Progression-free survival according to preoperative inflammatory markers

**Fig. 2:**
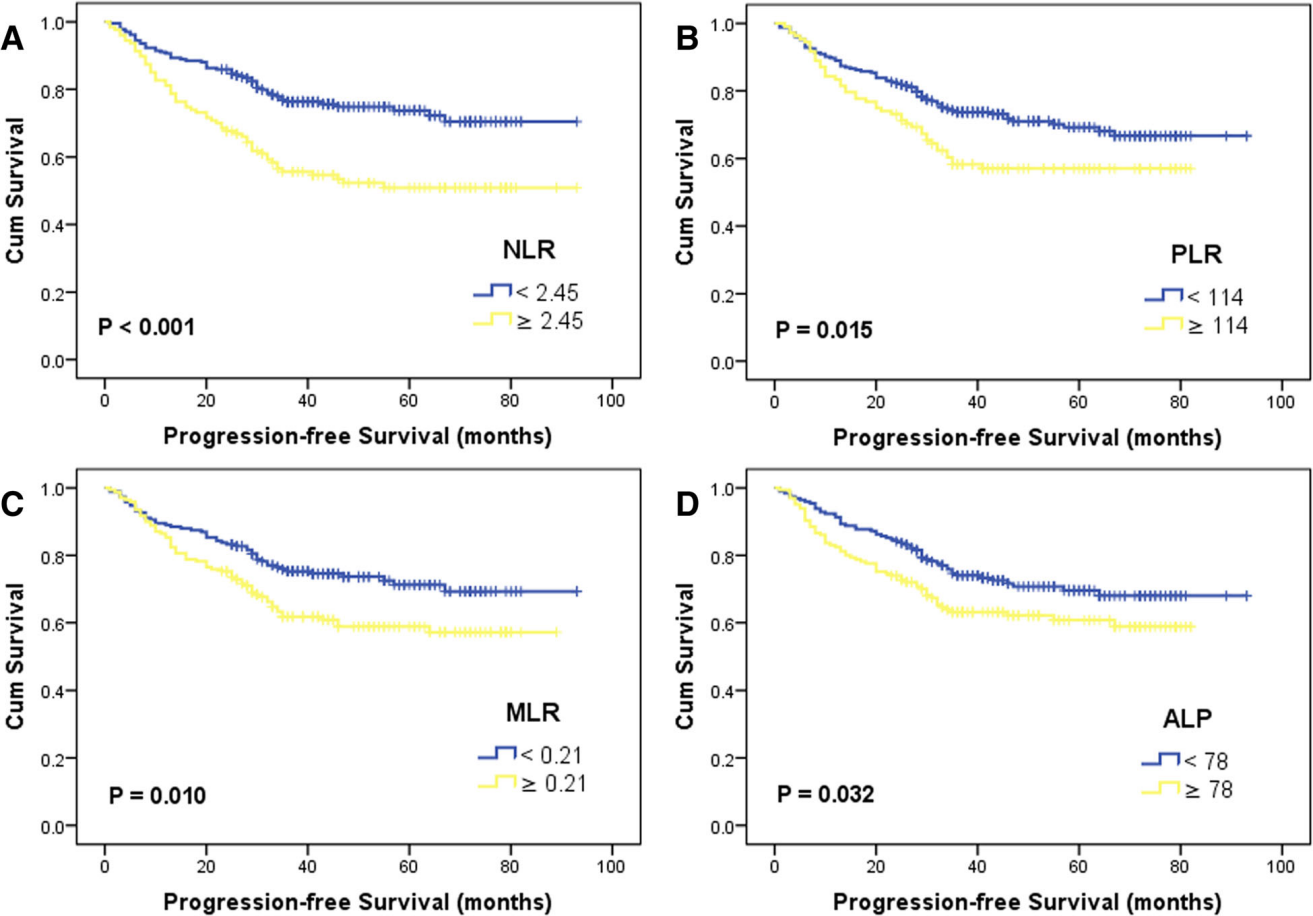
Kaplan-Meier survival curves for preoperative NLR **a** PLR **b** MLR **c** ALP **d** on progression-free survival

There were 117 patients had evidence of cancer progression. Forty, 15 and 7 patients developed local recurrence, cervical lymph nodes metastases and distant metastases, respectively. The 5-year PFS rates were 73.8% and 50.9% in two NLR groups (*p* < 0.001), 69.3% and 57.0% in two PLR groups (*p* = 0.015). Both high NLR (HR = 2.13, 95%CI: 1.49–3.07, *p* < 0.001) and PLR (HR = 1.58, 95%CI: 1.09–2.29, *p* = 0.016) were related with decreased PFS in univariate analysis (Fig. 2, Table 2). The HRs and 95%CIs were 1.61 (1.12–2.32, *p* = 0.011) of MLR groups and 1.48 (1.03–2.13, *p* = 0.034) of ALP groups, which reflected additional PFS benefiting from low MLR or ALP (Fig. 2, Table 2). Significant differences of survival curve were not found in the analysis of LDH (*p* = 0.451).

In univariate analysis, the patient’s age (*p* = 0.020), tumor location (*p* = 0.008, *p* < 0.001), T classification (*p* < 0.001), N classification (*p* < 0.001), TNM stage (*p* < 0.001), histology (*p* < 0.001) and laryngectomy (*p* < 0.001) reflected significant predictive values of PFS (Table 3). In the multivariate model, high NLR (HR = 1.52, 95%CI: 1.04–2.23, *p* = 0.029) and advanced TNM stage (HR = 3.41, 95%CI: 2.25–5.16, *p* < 0.001) remained independent predictors for poor PFS in LSCC patients (Table 4).

### Survival according to postoperative inflammatory markers

In terms of OS, the HRs and 95%CIs were 3.18 (2.08–4.86, *p* < 0.001), 3.13 (1.98–4.94, *p* < 0.001), 3.65 (2.40–5.56, *p* < 0.001) for NLR, PLR and MLR, respectively (Table 2, Additional file 3: Figure S2). The high NLR (HR = 2.18, 95%CI: 1.52–3.14, *p* < 0.001), PLR (HR = 1.85, 95%CI: 1.28–2.68, *p* = 0.001) and MLR (HR = 2.60, 95%CI: 1.79–3.79, *p* < 0.001) were associated with worse PFS (Table 2, Additional file 4: Figure S3). Neither ALP nor LDH were significant prognostic factors for OS and PFS. In multivariate analysis, the postoperative MLR was still significantly related with OS (HR = 2.02, 95%CI: 1.29–3.14, *p* = 0.002) and PFS (HR = 1.57, 95%CI: 1.05–2.34, *p* = 0.026) (Table 4).

## Discussion

Recruited by tumor cells, the tumor infiltrating neutrophils (TINs) can produce vascular endothelial growth factor (VEGF) and protease to promote the formation of tumor microenvironment and enhance tumor proliferation, angiogenesis, invasiveness and metastasis [18]. TINs also contribute to the epithelial-mesenchymal transition and infiltration growth of tumor cells [19, 20]. Cooperate with tumor-associated macrophages (TAMs), TINs can produce IL-6 and granulocyte colony-stimulating factor, activate the STAT3 signaling pathway to slow down neutrophil degranulation and enhance tumor proliferation [21, 22]. On the contrary, lymphocytes have an adverse effect on growth and maturation of tumor cells [23]. The elevated level of NLR means relative increase of neutrophils and decrease of lymphocytes, thereby increasing the risk of tumor recurrence and metastasis. In previous studies, the preoperative NLR was an independent predictor for OS, cancer-specific survival (CSS) and disease-free survival (DFS) in LSCC according to multivariate analyses [15, 24]. However, studies also indicated non-significant correlation between preoperative NLR and DFS [25], and high NLR was not independently related with poor OS in patients undergoing postoperative radiotherapy [9]. In our study, the preoperative NLR showed significant predictive role of OS and PFS, which supported preoperative NLR was an independent factor in the prognosis of patients with LSCC.

The PLR was also widely used in predicting survival outcome of cancer patients. The tumor cells can increase peripheral platelet count via thrombopoietin, IL-6 or leukemia inhibitory factor [26]. Then platelets can promote the growth and metastasis of tumor cells in turn. For instance, platelets can form platelets-tumor cell complexes to protect tumor cells from immune response. Activated platelets would secrete transforming growth factor-beta to suppress NK cells, and VEGF to promote tumor angiogenesis, which all contribute to the tumor proliferation and metastasis [27, 28]. Therefore, PLR was inversely relevant with patients’ survival and disease progression. The prognostic value of preoperative PLR in LSCC was less reported. The published studies concluded that low PLR was independently associated with better OS, CSS and DFS in multivariate analyses [9, 24]. However, we only found significant predictive role of preoperative PLR in univariate analyses. The PLR failed to be an independent prognostic biomarker, which indicated that preoperative NLR might be a better predictor for OS and PFS in LSCC.

In addition, the prognostic values of MLR, ALP and LDH were also estimated. The high preoperative ALP was prominently related with worse OS and PFS according to this univariate analyses. The increased ALP was often found in bone metastatic tumors or liver lesions, and showed prognostic function in several solid tumors such as metastatic prostate cancer [29]. The higher glycolysis of tumor tissue than normal tissue may result in elevated LDH [30]. However, preoperative LDH had no important effect on OS and PFS in this study. TAMs were derived from circulating monocytes, and the M2 phenotype of TAMs can stimulate tumor-cell proliferation, angiogenesis and metastasis via growth factors and angiogenic factors [31]. In our study, preoperative MLR showed prognostic value of OS and PFS in univariate analyses. However, all preoperative MLR, LDH and ALP were not independent predictors of OS and PFS, which urged more studies about MLR, ALP and LDH in LSCC patients.

There were few studies examining the prognostic role of post-treatment inflammatory markers in laryngeal cancer. Previous study indicated that post-treatment neutrophil count was related with OS and local control, but post-treatment NLR didn’t show prognostic value in oropharyngeal and laryngeal cancer undergoing radiotherapy [32]. However, the post-treatment NLR was an independent predictor of OS and PFS in advanced head and neck squamous cell carcinoma receiving chemoradiotherapy [33]. In this study, the high postoperative NLR, PLR and MLR were significantly associated with increased risk of death and tumor progression. And postoperative MLR remained an independent predictor of OS and PFS in LSCC patients.

Among the clinicopathological factors, TNM stage is widely used in estimation of prognosis and selection of standardized treatment plan. The present study also supported TNM stage as an independent prognostic factor of OS and PFS. However, whether age is an effective predictor of survival outcome remains controversial. The age at diagnosis was an independent predictor of OS in several previous articles [16, 34]. In contrast, researchers also found that there was no significant association between age and prognosis [3, 35]. Based on our study, age at diagnosis only had effect on prognosis in univariate analyses. Pathological T classification, N classification, histological differentiation, adjuvant therapy, medication and comorbidities failed to show significant results. Therefore, the independent prognostic value of clinicopathological factors and inflammatory markers requires further investigation.

There were several limitations in the present study. First of all, this study was a retrospective and single-center study that might cause selection bias, thus prospective and multi-center studies were needed to support the prognostic role of inflammatory markers. Secondly, although we added the significant parameters in Chi squared test into multivariate analyses, the various patients’ characteristics could be potential confounders that influence the survival outcome. Furthermore, since our study was limited to Chinese population, the preliminary results may not apply to other ethnic populations.

## Conclusions

In summary, elevated preoperative NLR, PLR, MLR and ALP were significantly associated with worse OS and PFS in LSCC patients undergoing surgical resection. In addition, preoperative NLR and postoperative MLR might be independent prognostic biomarkers for identifying survival outcome and cancer progression. The inflammatory markers were suggested to be novel and effective predictors with simple examination method. However, it still needs to be proved by further multi-center and large-scale prospective studies.

## Additional files

Additional file 1: **Figure S1.** The flowchart of patient selection. (TIF 4156 kb)

Additional file 2: **Table S1.** The mean value of preoperative inflammatory markers by patient’s clinicopathological characteristics (DOCX 27 kb)

Additional file 3: **Figure S2.** Kaplan-Meier survival curves for postoperative NLR (A), PLR (B), MLR (C), ALP (D) on overall survival. (TIF 4684 kb)

Additional file 4: **Figure S3.** Kaplan-Meier survival curves for postoperative NLR (A), PLR (B), MLR (C), ALP (D) on progression-free survival. (TIF 4684 kb)

## Abbreviations

ALP: alkaline phosphatase
AUC: area under the curve
CI: confidence intervals
CSS: cancer-specific survival
DFS: disease-free survival
HR: hazard ratio
LDH: lactate dehydrogenase
LSCC: Laryngeal squamous cell carcinoma
MLR: monocyte-to-lymphocyte ratio
NLR: neutrophil-to-lymphocyte ratio
OS: overall survival
PFS: progression-free survival
PLR: platelet-to-lymphocyte ratio
ROC: receiver operating curve
TAM: tumor-associated macrophages
TINs: tumor infiltrating neutrophils
