# Supplementary figures and images for: Survival and prognostic analysis of preoperative inflammatory markers in patients undergoing surgical resection for laryngeal squamous cell carcinoma
Source: BMC Cancer. 2018 Aug 13;18:816. doi: 10.1186/s12885-018-4730-x (PMC6090788; doi:10.1186/s12885-018-4730-x)

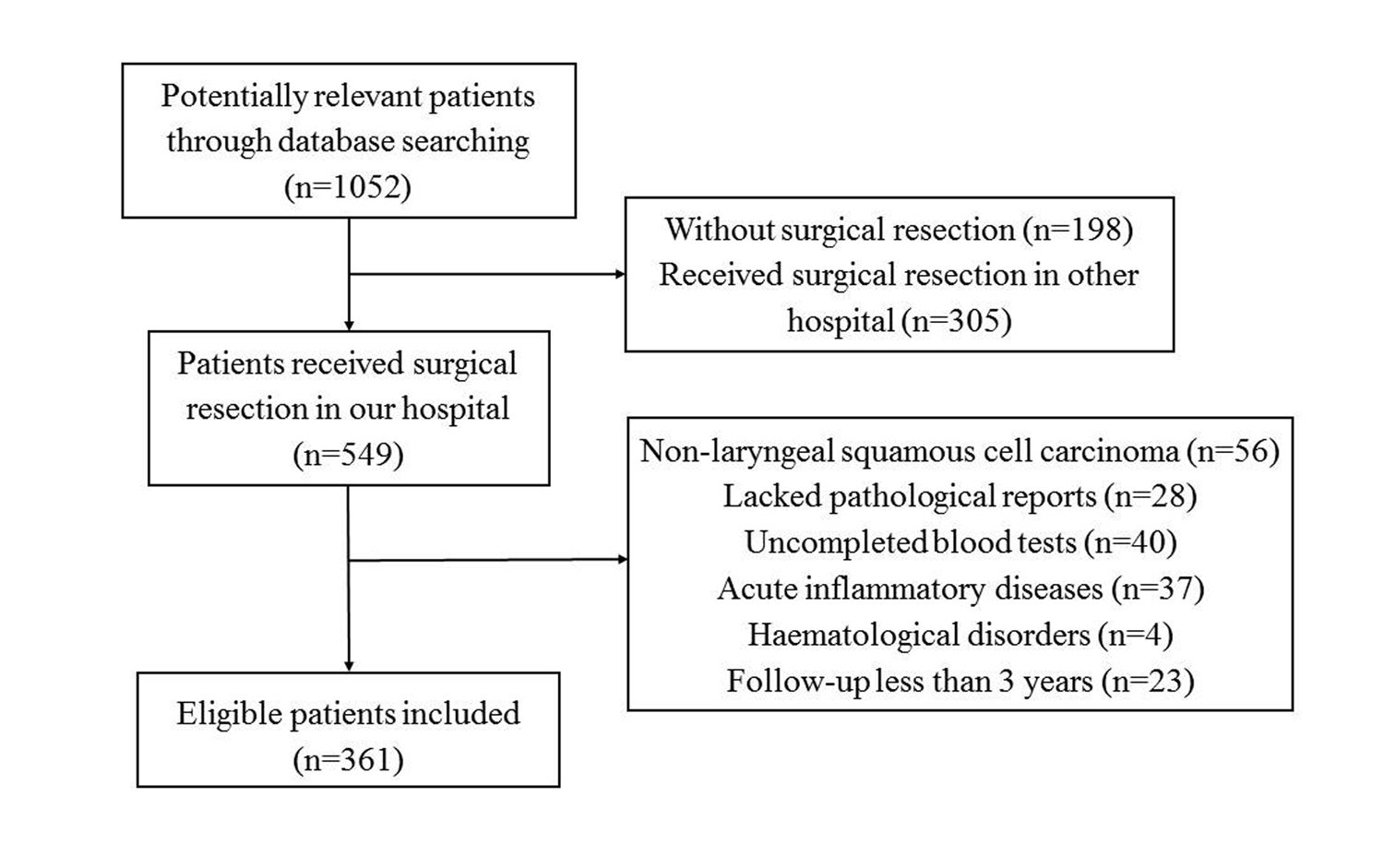

Supplement: Supplementary file 1 — Figure S1. The flowchart of patient selection. (TIF 4156 kb) [file 12885_2018_4730_MOESM1_ESM.tif]

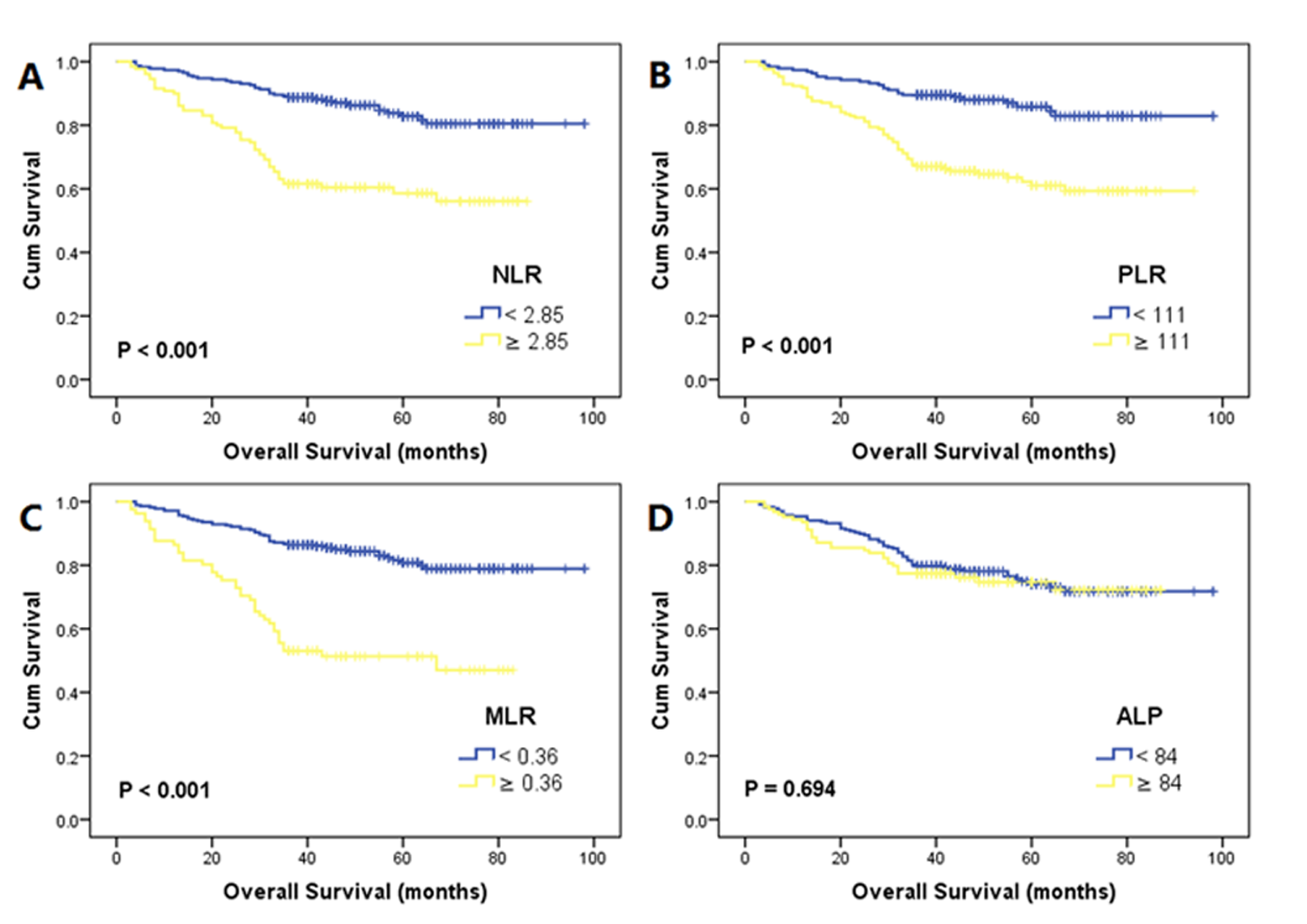

Supplement: Supplementary file 3 — Figure S2. Kaplan-Meier survival curves for postoperative NLR (A), PLR (B), MLR (C), ALP (D) on overall survival. (TIF 4684 kb) [file 12885_2018_4730_MOESM3_ESM.tif]

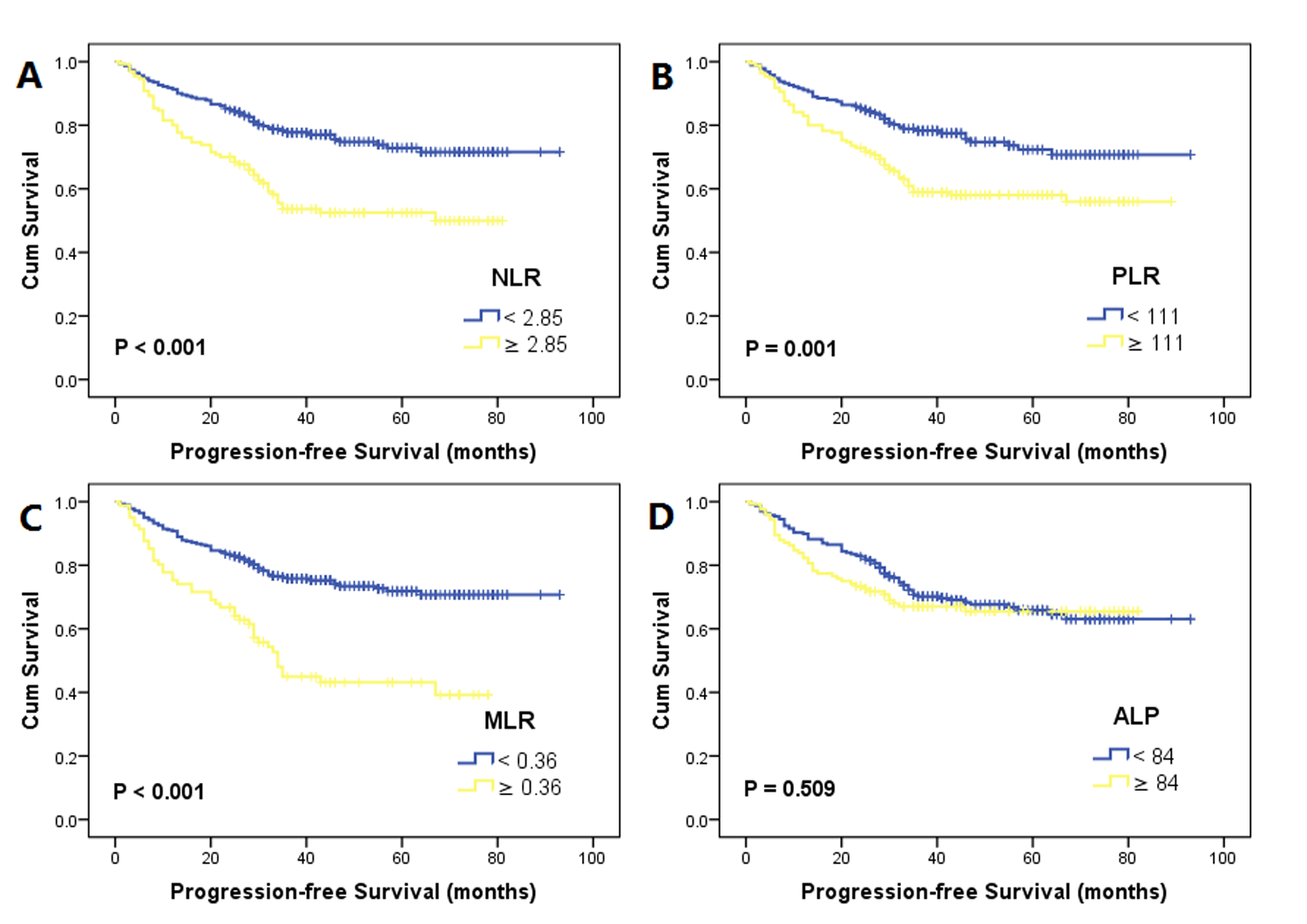

Supplement: Supplementary file 4 — Figure S3. Kaplan-Meier survival curves for postoperative NLR (A), PLR (B), MLR (C), ALP (D) on progression-free survival. (TIF 4684 kb) [file 12885_2018_4730_MOESM4_ESM.tif]
