# Supplementary material for: Survival and prognostic analysis of preoperative inflammatory markers in patients undergoing surgical resection for laryngeal squamous cell carcinoma
Source: BMC Cancer. 2018 Aug 13;18:816. doi: 10.1186/s12885-018-4730-x (PMC6090788; doi:10.1186/s12885-018-4730-x)
Supplement: Supplementary file 2 — Table S1. The mean value of preoperative inflammatory markers by patient’s clinicopathological characteristics (DOCX 27 kb) [file 12885_2018_4730_MOESM2_ESM.docx]

**Table S1. The mean value of preoperative inflammatory markers by patient’s clinicopathological characteristics**

| **Variables** | **Parameters** | **n** | **NLR** | ***p*** | **PLR** | ***p*** | **MLR** | ***p*** | **ALP** | ***p*** | **LDH** | ***p*** |
| --- | --- | --- | --- | --- | --- | --- | --- | --- | --- | --- | --- | --- |
|  |  |  | **(mean±SD)** |  | **(mean±SD)** |  | **(mean±SD)** |  | **(mean±SD)** |  | **(mean±SD)** |  |
| Age (year) | < 60 | 167 | 2.31±1.19 | 0.168 | 100.15±53.10 | 0.126 | 0.235±0.114 | 0.687 | 79.88±22.91 | 0.148 | 167.17±33.77 | 0.018* |
|  | ≥ 60 | 194 | 2.53±1.91 |  | 112.34±94.74 |  | 0.240±0.138 |  | 76.66±18.54 |  | 177.04±43.82 |  |
| Gender | Male | 353 | 2.41±1.58 | 0.511 | 137.49±99.67 | 0.262 | 0.238±0.127 | 0.683 | 78.09±20.76 | 0.720 | 172.67±40.04 | 0.537 |
|  | Female | 8 | 3.13±2.93 |  | 106.00±77.93 |  | 0.220±0.169 |  | 80.75±21.06 |  | 163.88±22.71 |  |
| Tumor location | Supraglottic | 280 | 2.33±1.45 | 0.036* | 101.93±72.67 | 0.047* | 0.232±0.117 | 0.137 | 77.80±20.31 | 0.049* | 172.06±41.05 | 0.926 |
|  | Glottic | 70 | 2.65±1.50 |  | 119.01±62.80 |  | 0.254±0.133 |  | 77.17±21.90 |  | 174.14±36.55 |  |
|  | Subglottic | 11 | 3.43±4.25 |  | 149.76±207.48 |  | 0.293±0.277 |  | 93.18±20.35 |  | 172.36±24.67 |  |
| T classification | T1 | 115 | 2.14±1.00 | <0.001* | 97.00±55.25 | 0.001* | 0.214±0.095 | 0.003* | 76.51±17.50 | 0.138 | 171.01±34.43 | 0.590 |
|  | T2 | 126 | 2.26±1.37 |  | 97.18±70.31 |  | 0.230±0.117 |  | 76.52±20.36 |  | 170.2±35.95 |  |
|  | T3 | 68 | 2.62±1.88 |  | 110.04±76.16 |  | 0.254±0.147 |  | 82.46±25.20 |  | 174.84±56.94 |  |
|  | T4 | 52 | 3.24±2.44 |  | 146.87±121.43 |  | 0.288±0.168 |  | 80.73±21.35 |  | 178.25±31.80 |  |
| N classification | N0 | 320 | 2.37±1.39 | 0.223 | 101.90±67.83 | 0.047* | 0.232±0.114 | 0.096 | 78.11±20.46 | 0.912 | 173.12±41.14 | 0.390 |
|  | N1-N2 | 28 | 2.92±2.81 |  | 144.20±130.45 |  | 0.286±0.201 |  | 78.94±23.08 |  | 167.44±26.15 |  |
| TNM stage | I | 114 | 2.13±1.01 | 0.001* | 96.20±54.81 | 0.001* | 0.213±0.095 | 0.005* | 76.68±17.49 | 0.051 | 171.22±34.51 | 0.756 |
|  | II | 118 | 2.27±1.40 |  | 97.00±71.44 |  | 0.231±0.120 |  | 75.66±19.94 |  | 170.51±36.92 |  |
|  | III | 71 | 2.63±1.85 |  | 108.98±75.02 |  | 0.252±0.145 |  | 83.85±25.27 |  | 174.37±55.75 |  |
|  | IV | 58 | 3.11±2.35 |  | 144.30±116.74 |  | 0.282±0.161 |  | 79.14±21.26 |  | 176.62±31.06 |  |
| Histology | Well | 135 | 2.36±1.82 | 0.152 | 105.60±97.08 | 0.222 | 0.232±0.130 | 0.359 | 75.60±20.58 | 0.106 | 171.24±33.85 | 0.883 |
|  | Moderate | 159 | 2.34±1.42 |  | 101.52±63.33 |  | 0.234±0.129 |  | 78.68±19.54 |  | 172.86±47.68 |  |
|  | Poor | 67 | 2.78±1.59 |  | 121.22±70.09 |  | 0.258±0.118 |  | 82.03±23.30 |  | 174.04±28.97 |  |
| Laryngectomy | Partial | 287 | 2.21±1.22 | 0.001* | 97.33±62.83 | 0.001* | 0.221±0.103 | <0.001* | 77.32±20.90 | 0.135 | 169.68±33.18 | 0.054 |
|  | Total | 74 | 3.25±2.48 |  | 143.05±114.77 |  | 0.301±0.182 |  | 81.36±19.91 |  | 183.32±56.67 |  |
| Radiotherapy | No | 279 | 2.41±1.52 | 0.702 | 104.41±72.38 | 0.306 | 0.237±0.118 | 0.890 | 79.00±21.44 | 0.151 | 173.97±40.92 | 0.188 |
|  | Yes | 82 | 2.49±1.92 |  | 114.50±96.27 |  | 0.240±0.157 |  | 75.26±17.99 |  | 167.39±35.17 |  |
| Chemotherapy | No | 335 | 2.35±1.42 | 0.063 | 103.06±67.92 | 0.114 | 0.233±0.114 | 0.198 | 78.49±20.92 | 0.264 | 172.39±40.50 | 0.883 |
|  | Yes | 26 | 3.53±3.09 |  | 153.57±156.37 |  | 0.295±0.236 |  | 73.77±18.12 |  | 173.58±28.83 |  |
| Medication | No | 314 | 2.44±1.67 | 0.743 | 107.25±80.26 | 0.733 | 0.238±0.132 | 0.842 | 78.02±20.68 | 0.752 | 171.32±40.41 | 0.153 |
|  | Yes | 47 | 2.36±1.21 |  | 103.05±65.39 |  | 0.234±0.095 |  | 79.04±21.33 |  | 180.21±34.33 |  |
| Comorbidities | No | 274 | 2.42±1.48 | 0.763 | 106.85±73.94 | 0.950 | 0.236±0.123 | 0.666 | 76.93±20.10 | 0.048* | 172.10±41.66 | 0.751 |
|  | Yes | 87 | 2.48±1.99 |  | 106.24±91.57 |  | 0.243±0.142 |  | 81.98±22.35 |  | 173.66±33.15 |  |

Abbreviations: NLR neutrophil-lymphocyte ratio, PLR platelet-lymphocyte ratio, MLR monocyte-lymphocyte ratio, ALP alkaline phosphatase, LDH lactate dehydrogenase.

^*^ Statistically significant *p* < 0.05
